# Comparison of quality, birth outcomes, and service utilization between health facilities with and without maternity waiting homes in Liberia

**DOI:** 10.1016/j.midw.2021.103235

**Published:** 2022-02

**Authors:** Rachel Horton, Haeun Lee, Joseph E. Perosky, Alphonso Kofa, Jody R. Lori

**Affiliations:** a Undergraduate Student, University of Michigan, School of Nursing, 400 North Ingalls, Ann Arbor, MI 48104, United States; b Graduate Student, University of Michigan, School of Nursing, 400 North Ingalls, Ann Arbor, MI 48104, United States; c Resident Physician, Department of Obstetrics, Gynecology and Reproductive Biology, Michigan State University, 400 North Ingalls, Ann Arbor, MI 48104, United States; d Director - Bong County Health, Suakoko, Liberia; e Professor & Associate Dean for Global Affairs, University of Michigan, School of Nursing, 400 North Ingalls, Ann Arbor, MI 48104, United States

**Keywords:** Maternity waiting homes, Birth outcomes, Perinatal care, Quality, Service utilization, Liberia

## Abstract

**Objective:**

1) To assess the quality of health facilities associated with functional Maternity Waiting Homes and health facilities without functional maternity waiting homes in Liberia. 2) To examine birth outcomes and care utilization amongst health facilities with and without functional maternity waiting homes in Liberia.

**Design:**

Secondary analysis design using data from a facility capacity checklist and Liberia's Health Management Information System.

**Setting:**

71 health facilities associated with functional maternity waiting homes and 14 health facilities without functional maternity waiting homes across 14 counties of Liberia.

**Participants:**

No human participants were used in this study.

**Methods:**

Independent *t*-test, Pearson chi-square test, and logistic regression were performed to assess quality, birth outcomes, and service utilization between health facilities with and without functional maternity waiting homes.

**Findings:**

The overall health facility quality was not significantly different between health facilities associated with functional maternity waiting homes and those without. However, health facilities with functional maternity waiting homes had better infection control with the presence of soap and sharps boxes. Health facilities with functional maternity waiting homes were also more likely to have parenteral oxytocic drugs and were better able to perform assisted vaginal deliveries. The presence of functional maternity waiting homes were not significantly associated with health facility quality, birth outcomes, or care utilization.

**Conclusion and implications:**

Health facilities with functional MWHs were better prepared to prevent infection and manage complicated deliveries. This study further highlights specific areas for quality improvement amongst these health facilities, including labor complications management.

## Introduction

Maternal mortality is a major public health issue that disproportionately affects low-income countries with approximately 295,000 women dying during or after childbirth worldwide annually (World Health Organization, 2019). In Liberia, the most recent maternal mortality ratio is estimated to be 725 deaths per 100,000 live births (Bjegovic-Mikanovic et al., 2018; African Health Observatory, n.d.) Many efforts have been made to decrease maternal mortality in Liberia, including the implementation of Maternity Waiting Homes (MWHs) (Bekele et al., 2019). A MWH is a dwelling place near a health facility (HF) where pregnant women can stay prior to delivery or in the postpartum period. By directly addressing the barriers to reaching and receiving care, MWHs increase antenatal care (ANC) and postnatal care (PNC) visits as well as delivery with skilled birth attendants (SBA) at healthcare facilities (Bailey et al., 2009; Lori et al., 2014; Lori et al., 2019; Henry et al., 2017; Braat et al., 2018; Bekele et al., 2019). In Liberia, MWHs were established in Bong County between 2010 and 2014 through a matched cohort study to assess the impact of these MWHs on maternal and neonatal health outcomes (Lori et al., 2014). While there was a gap in research between 2014 and 2018 due to Liberia's Ebola Outbreak, MWHs continued to scale-up across the country via various mechanisms (Perosky et al., 2020).

According to The Lancet Commission on Quality, improved accessibility is not sufficient and high-quality healthcare systems are the backbone of outcome improvement (Kruk et al., 2018 ). While MWHs help women overcome the distance barrier to accessing care, the quality of the health facilities associated with the MWHs may be suboptimal to provide quality care to improve maternal and infant health outcomes. In 2018, researchers found that of the 8.6 million deaths in low-income countries associated with health care delivery, 5 million deaths were due to poor quality care, compared to 3.6 million deaths due to lack of care utilization (Kruk et al., 2018). In Liberia 4298 deaths per 100,000 total deaths were related to poor quality of care compared to 3954 deaths related to non-utilization of care (Kruk et al., 2018).

One way to assess a HF's quality is to assess the HF's ability to perform Basic Emergency Obstetric and Newborn Care (BEmONC) functions. MWHs are implemented in close proximity to BEmONC facilities, which in theory, are equipped with resources and staff to provide life-saving interventions to reduce maternal and newborn morbidity and mortality (Bailey et al., 2009; Otolorin et al., 2015). To qualify as a basic BEmONC facility, trained staff must be available 24 h a day, seven days per week, meet the needs of the population, and perform all seven signal functions within a three-month period (Bailey et al., 2009). The seven signal functions of basic BEmONC include: (1) parenteral antibiotics administration, (2) parenteral uterotonics administration, (3) parenteral anticonvulsants administration, (4) manual removal of retained placenta, (5) removal of retained products of conception with manual vacuum aspiration, (6) assisted vaginal delivery, and (7) basic neonatal resuscitation (Bailey et al., 2009).

To date, there is no standardized tool for assessing basic BEmONC quality. The primary method of assessing basic BEmONC quality is through evaluation of the seven signal functions (Mahato et al., 2017; Ntambue et al., 2017; Solnes Miltenburg et al., 2017). Considering the World Health Organization's (WHO) recent evaluation of BEmONC services available and utilized in HFs internationally excluded Liberia, this study aims to provide crucial insights regarding facility quality and provision of BEmONC services amongst Liberian HFs (Bailey et al., 2009).

Therefore, the aim of this study is to examine the quality of health facilities associated with functional MWHs and those without functional MWHs. Additionally, the study examined the difference in delivery outcomes and service utilization between health facilities with and without functional MWHs. For the purpose of this study, functional was defined as open and providing space for pregnant women awaiting delivery. Non-functional was defined as currently under construction, construction started but ceased prior to opening, repurposed for other use, or abandoned.

## Materials and methods

### Overview

This study examined two data sets:1) a facility capacity checklist and 2) Liberia's Health Management Information System (HMIS) data. The facility capacity checklist was developed in our parent study for a nation-wide assessment of MWHs sustainability of the 119 newly formed MWHs in Liberia (Lori et al., 2020). Information regarding facility infrastructure and its ability to perform BEmONC signal functions were extracted for the study. The HMIS data were obtained from the Bong County Health Department and used to extract maternal and infant health outcomes and service utilization. We matched the two data sets by HF name and conducted independent *t*-test, Pearson chi-square test, and logistic regression to assess quality, birth outcomes, and service utilization between health facilities with and without maternity waiting homes.

Ethical approval for the parent study was obtained from the University of Michigan Health Sciences and Behavioral Sciences Institutional Review Board (IRB) and from the University of Liberia Pacific Institute for Research and Evaluation.

### Data

The facility capacity checklist data were collected for the parent study between December 2017 to June 2018 to examine the effectiveness, sustainability, and scalability of 85 out of the 119 MWHs in Liberia (Lori et al., 2020). The facilities in the study were selected through purposive sampling as only health facilities associated with MWH in Liberia were chosen. Of the 85 that were surveyed, 71 were functional and open to the public.

Inclusion criteria required that the facilities were: 1) located within catchment areas of each MWH; 2) staffed with BEmONC trained providers for 24 h per day, seven days per week; and 3) located within a 2-hour travel time from the comprehensive EmONC facility. The original data were collected at the facility level; semi-structured interviews were conducted by one US and one Liberian researcher, and two Liberian research assistants with the officer in charge of each facility. Facility logbooks were reviewed with permission granted through telephone and in-person interactions with the county health directors. The data contains information about facility infrastructure, availability of services, protocols, staff, medicines, supplies, and the seven BEmONC signal functions of for 85 HFs associated with MWH across 14 counties of Liberia. The seven signal functions of BEmONC were also assessed according to the WHO standard (Bailey et al., 2009; Solnes Miltenburg et al., 2017). For this study, data related to facility infrastructure, staffing, and the seven BEmONC signal functions were extracted for analysis.

The HMIS is comprised of data designed and collected by the Liberian Ministry of Health to improve all levels of healthcare delivery through better planning and management across counties. County health teams are responsible for collecting data from all health and social welfare activities occurring in hospitals, health centers, and clinics under their supervision. Birth outcome variables (maternal mortality and stillbirths), as well as service utilization variables (ANC, PNC, and facility-based deliveries), were extracted from the 2017–2018 HMIS data.

### Data analysis

Data were analyzed using Stata 15.0 (StataCorp, College Station, TX, USA). Descriptive analysis of the facility checklist data was conducted using independent t-tests and Pearson chi-square tests to understand the status of BEmONC associated with both functioning and non-functioning MWHs in Liberia.

The facility capacity checklist served as our indicator of HF quality. Facility capacity checklist data were categorized into three composite score variables: a composite infection control score, a composite BEmONC score, and an overall composite quality score. The composite infection control score consisted of the availability of: 1) water, 2) soap, 3) gloves, 4) sharps box, and 5) improved source of water (improved: piped/ municipal water, hand pump/borehole vs. unimproved: well, or river). The maximum infection control composite score was 5, indicating that all components of the composite score were present.

The composite BEmONC score consisted of the availability of: 1) parenteral antibiotics for maternal infection, 2) parenteral oxytocic drugs for hemorrhage, 3) parenteral magnesium sulfate for eclampsia, and the ability of the providers to perform 4) manual placenta removal, 5) removal of retained products of conception, 6) assisted vaginal delivery, and 7) newborn resuscitation with bag and mask of non-breathing baby, with a maximum score of 7.

The overall composite quality score combined variables from the composite infection score, composite BEmONC score, presence of electricity and a backup generator, and presence of skilled providers.

A descriptive analysis examining the two groups of HFs was also performed using the HMIS data variables. These variables included: 1) ANC services, 2) facility-based deliveries, 3) maternal deaths, 4) stillbirths, and 5) PNC services. Independent t-tests were used to understand birth outcomes and service utilization associated with health facilities with and without functional MWHs in Liberia. Due to unequal sized groups, data were normalized for catchment population (number of outcome variable divided by the catchment population and multiplied by 10,000).

## Results

Of the 85 HFs for which facility capacity checklist data were obtained, 71 HFs (83.53%) were associated with functional MWHs. Table 1 shows a comparison of quality between HFs associated with functional MWHs and HFs associated with non-functional MWHs. Of the HFs associated with MWHs, 90.14% had electricity and 46.48% had a backup generator, as compared to 78.57% and 28.57%, respectively, of HFs associated with non-functional MWHs. Both groups of facilities had similar employment rates of skilled providers and providers working on the day of data collection.

**Table 1:** Facility status between Health Facilities (HF) with open and functioning Maternity Waiting Homes (MWHs) and HFs without open and running MWHs.

	HF with open and functioning MWHs	HF without open and functioning MWHs	P-value
Facilities, n (%)	71 (83.53)	14 (16.47)	
**Composite quality score**^a^**mean (SD)**	11.14 (1.74)	10.21 (2.04)	0.08
Electricity, n (%)	64 (90.14)	11 (78.57)	0.21
Backup generator, n (%)	33(46.48)	4 (28.57)	0.21
**Composite infection control Score**^b^**mean (SD)**	4.49 (0.67)	4.07 (0.91)	0.047*
Water, n (%)	65 (91.55)	13 (92.86)	0.87
Soap, n (%)	58 (81.69)	7 (50.00)	0.01*
Gloves, n (%)	62 (87.32)	14 (100.00)	0.15
Sharps box, n (%)	71 (100.00)	13 (92.86)	0.02*
Source of water, n (%)			0.18
Piped/ Municipal water	6 (8.45)	0 (0)	
Hand pump/ Borehole	57 (80.28)	10 (71.43)	
Well	4 (5.63)	1 (7.14)	
River	4 (5.63)	3 (21.43)	
**Skilled provider, mean (SD)**			
Employed	4.14 (3.94)	3.5 (1.74)	0.55
Working today	2.92 (1.95)	2.92 (1.85)	0.99
			
**Composite basic EmONC score**^c^**mean (SD)**	4.30 (1.19)	4.14 (1.23)	0.63
Parenteral antibiotics for maternal infection	13 (18.84)	3 (21.43)	0.82
Parenteral oxytocic drugs for hemorrhage	64 (90.14)	10 (71.43)	0.03*
Parenteral Magnesium sulfate for eclampsia	49 (69.01)	11 (78.57)	0.51
Manual removal of the placenta	64 (90.14)	13 (92.86)	0.85
Removal of retained products of conception	34 (47.89)	8 (57.14)	0.59
Assisted vaginal delivery	18 (25.35)	0 (0.00)	0.03*
Resuscitation with bag and mask of non-breathing baby	64 (90.14)	13 (92.86)	0.86

Independent *t*-test was conducted for continuous variables and Pearson chi-square test was conducted for categorical variables.

a : Composite quality score: Highest value possible: 15. Consisted of the availability of 1) electricity, 2) backup generator, 3) water, 4) soap, 5) gloves, 6) sharps box, 7) improved source of water (Piped/ municipal water or Hand pump/ Borehole), 8) skilled care provider working the day of data collection, 9) parenteral antibiotics, 10) parenteral oxytocic, 11) parenteral Magnesium sulfate, ability to perform 12) manual removal of the placenta 13) removal of retained products of conception 14) assisted vaginal delivery, and15) newborn bagging.

b : Composite infection score: Highest value possible: 5. Consisted of the availability of 1) water, 2) soap, 3) gloves, 4) sharps box, 5) improved source of water (Piped/ municipal water or Hand pump/ Borehole).

c : Composite basic Emergency Obstetric Care Services (EmONC) score: Highest value possible: 7. Consisted of the availability of 1) parenteral antibiotics, 2) parenteral oxytocic, 3) parenteral Magnesium sulfate, and the ability to perform 4) manual removal of the placenta 5) removal of retained products of conception 6) assisted vaginal delivery, and 7) newborn bagging. *p<0.05.

The three types of composite scores (composite quality score, composite infection score, composite BEmONC score) are also depicted in Table 1. The composite quality scores of HFs with functional MWHs is 11.14 (out of 15) and 10.21 for HFs non-functional MWHs. There was no significant difference between the two types of HFs in their composite quality score.

For composite infection control score, HFs with functional MWHs averaged 4.49 (out of 5) and HFs non-functional MWHs averaged 4.07, with a significant difference between the two types of HFs (*p*<0.05). When each of the variables of the composite infection control score is further examined, HFs associated with functional MWHs had greater presence of soap (*p*<0.01) and sharps boxes (*p*<0.05). Compared to 81% of the HFs with functional MWHs, only 50% of the HFs with non-functional MWHs had soap. Additionally, while all of the HFs with functional MWHs had sharps box, 93% of the HFs with non-functional MWHs had them.

For composite BEmONC scores, HFs with functional MWHs averaged 4.30 (out of 7) and HFs with non-functional MWHs averaged 4.14, with no significant difference between the two types of HFs. However, when each of the variables of the composite BEmONC score is examined individually, there is a significant difference between the facilities with and without functional MWHs in the availability of parenteral oxytocic drugs for hemorrhage (90% vs. 71%; *p*<0.05) and assisted vaginal delivery (25% vs. 0%; *p*<0.05).

Table 2 illustrates a comparison of maternal mortality and stillbirths and the reproductive health service utilization between HFs associated with functional MWH and those without. Birth outcomes, including stillbirth and maternal death, were not significantly different between the two types of HFs. Furthermore, service utilization, including delivery at health facility with skilled provider, attending four or more antenatal care visits, attending postnatal care within 24 h, and at 6 weeks were examined and showed no significant differences between HFs with functional MWHs and those non-functional MWHs.

**Table 2:** Maternal and neonatal outcomes and accessed services between Health Facilities (HF) with open, functional Maternity Waiting Homes (MWHs) and HFs without open, functional MWHs.

	HF with open and functioning MWHs	HF without open and functioning MWHs	P-value
Facilities, n (%)	71 (83.53)	14 (16.47)	
Still birth, mean (SD)	6.74 (13.93)	4.09 (8.90)	0.49
Maternal death, mean (SD)	0.51 (1.50)	0.49 (1.06)	0.97
Delivery at health facility with skilled provider, mean (SD)	802.06 (1013.94)	617.93 (479.15)	0.50
Four or more antenatal care, mean (SD)	833.09 (1656.86)	493.49 (522.82)	0.45
Postnatal care within 24 h, mean (SD)	342.94 (623.84)	275.05 (302.78)	0.69
Postnatal care at 6 weeks, mean (SD)	552.41 (585.98)	459.16 (469.89)	0.57

Independent *t*-test conducted.

Normalized by var/catchment population *10,000.

## Discussion

This study aimed to inspect the quality of HFs associated with and without functional MWHs in Liberia and the subsequent influence on birth outcomes and service utilization. We found no significant differences between the overall quality, service utilization, or birth outcomes between HFs associated with functional MWHs and those without non-functional MWH. However, HFs associated with functional MWH were better equipped with resources to prevent infection and manage delivery complications. Additionally, our study presents important insights regarding the overall health facilities both with and without functioal MWHs in Liberia.

A high percentage of HFs in our study had electricity, 90.14% of the HFs with functional MWHs had electricity and 46.48% had backup generator. 78.57% of the HFs with non-functional MWHs had electricity and 28.57% had backup generator. Considering that a study by Adair-Rohani and colleagues (2013) showed that of 11 major sub-Saharan African countries, roughly 25% of HFs had no access to electricity the presence of electricity and backup generators in our sample seems to be high especially those associated with functional MWHs.

Composite quality scores (11.14 and 10.21 out of 15) as well as composite infection control scores (4.49 and 4.07 out of 5) were high in our study. However, these data highlighted that HFs with non-functional MWHs had a limited supply of soap and sharps boxes. Focusing on these two infection control items could serve as a starting point for improving the quality at this subset of HFs.

The composite BEmONC scores were low, averaging 4.30 and 4.14 (out of 7) respectively. This result shows that both groups have room for improvement within the domain of BEmONC services. The BEmONC indicators with the most room for growth include presence of parenteral antibiotics for maternal infection and provider skills in assisted vaginal delivery. These results align with findings from other facilities in low-income countries that are consistently unable to meet BEmONC standards as defined by WHO (Mahato et al., 2017; Tembo et al., 2017). In other studies, barriers to delivering BEmONC services include a lack of supplies, drugs, equipment, and trained staff (Mahato et al., 2017; Tembo et al., 2017). We found these same barriers in our study, including limited parenteral antibiotics and trained staff to provide vacuum assisted deliveries, which may hinder these HFs from responding to maternal complications and thus impact maternal and infant health outcomes.

Health facilities in our study had on average three to four skilled providers on staff with nearly three staff available at all facilities in our sample. Shortages of health care workers are critical in many low-income countries. WHO defines a critical shortage as less than 2.28 health workers per 1000 population (Mohr, 2006). At the time of data collection, the HFs in this study were staffed with nearly three healthcare workers on average, however, the catchment populations of all HFs in this study were over 1000 population. Our results do not indicate critical shortages across the HFs, however, more research is needed to determine the level of staff training and preparedness. Discrepancies in the scope of practice between different providers and lack of training have been found to be barriers to delivering BEmONC services (Solnes Miltenburg et al., 2017). Another prevalent issue is the reluctance by providers to perform BEmONC services that are more procedural in nature- such as vacuum-assisted deliveries (Ntambue et al., 2017; Tembo et al., 2017). Continued strategies to improve quality must be coupled with country wide initiatives to increase the healthcare workforce with specialized training programs in the provision of BEmONC services.

Based on the data, the presence of functional MWHs did not make a significant difference in the rates of maternal deaths, stillbirths, delivery by skilled attendants, or utilization of ANC or PNC. Additionally, these outcomes did not differ when HFs were further segmented by their composite quality scores. We anticipated that the presence of MWHs, especially high quality HFs and MWHs, would reduce the rates of perinatal mortality and improve service utilization. However, this was not reflected in our data.

It is important to consider that our data also did not identify significant differences in quality between HFs with functional MWHs and those without. The functional MWHs may be readily available for public use, however the related HFs continue to operate with a similar quality to the HFs associated with non-functional MWHs. Other studies have found that, despite having access to care and essential treatments, maternal mortality remains higher than expected in many countries (Akachi and Kruk, 2017). Furthermore, increasing rates of facility-based deliveries have not been matched by an equivalent reduction in maternal mortality, suggesting inadequacies in quality of care (World Health Organization, 2016).

The scale up of MWHs across Liberia seen in our parent study creates more opportunities for women to receive ANC services and facility-based deliveries with the presence of BEmONC resources. However, this scale up alone is not sufficient to improve maternal and neonatal mortality and service utilization without the provision of consistent, high-quality care. Moving forward, additional emphasis on reaching a standard of quality across all Liberian HFs is needed to see nationwide improvements of perinatal outcomes and service utilization.

## Limitations

This study has several limitations. First, purposive sampling was employed, thus not representing equal distribution of health facilities throughout the 14 counties of Liberia. Furthermore, due to the nature of the study design, the group sizes between HFs with and without a functional MWH are unbalanced. However, a strength of this study is the wide and diverse sample of at least one MWH from 14 out of the 15 counties in Liberia.

Second, we acknowledge there are more variables related to HF quality beyond the ones examined in this study. However, we utilized some of the key variables related to HF quality including facility infrastructure, staffing, services, and resource provided at the facility.

Lastly, because there is not a standardized tool to assess BEmONC signal functions, we extracted the corresponding variables from the facility capacity checklist. Considering the importance of BEmONC functions related to pregnancy and childbirth, there is a significant need for a standardized tool to collect data related to these functions. However, the method we utilized is often used in the literature (Bailey et al., 2009; Mahato et al., 2017; Ntambue et al., 2017; Solnes Miltenburg., 2017; Tembo et al., 2017). Despite these limitations, this study provides important insights regarding the overall quality of HFs associated with and without functional MWHs and identifies the strength and needs to further improve Liberia's HF quality.

## Conclusion

The overall HF quality was not significantly different between those associated with functional MWHs and those without. However, HFs with open and functional MWHs had better infection control and were more prepared to manage complicated deliveries. The culmination of these data highlights specific areas for improvement. Specifically, among the HFs examined in this study, increased availability of soap, sharps boxes, antibiotics, and adequately trained staff and supplies to perform vacuum assisted deliveries show significant room for improvement. To expand upon and sustain HFs and MWHs in the coming years, resources and energy should be directed towards these domains.

Maternity waiting homes are designed to overcome the distance barrier to accessing maternal health services (Lori et al., 2019). However, poor quality at HFs associated with the MWHs poses a significant barrier to the utilization of the important perinatal services to improve maternal and infant health outcomes. Therefore, it is not surprising that our study found little significant difference in quality between HFs with open and functioning MWHs and those without as well as no significant difference in birth outcomes and service utilization. As emphasized by the Lancet Commission on Quality, simply increasing accessibility to care cannot positively impact health outcomes alone; quality care must also be provided (Kruk et al., 2018). Future research is needed on successful strategies to improve the quality of care at HFs associated with MWHs as part of the comprehensive package of services to improve maternal and newborn outcomes in low resource settings.

## Ethical approval

Ethical approval for the parent study was obtained from the University of Michigan Health Sciences and Behavioral Sciences Institutional Review Board (IRB) and from the University of Liberia Pacific Institute for Research and Evaluation.

## Funding sources

This work was supported by the 10.13039/100000865Bill and Melinda Gates Foundation (OPP1170983). The funders had no role in study design, data collection and analysis, decision to publish, or preparation of the manuscript.

## CRediT authorship contribution statement

**Rachel Horton:** Conceptualization, Validation, Writing – original draft. **Haeun Lee:** Conceptualization, Methodology, Software, Formal analysis, Writing – review & editing. **Joseph E. Perosky:** Resources, Validation, Writing – review & editing. **Alphonso Kofa:** Resources, Validation. **Jody R. Lori:** Resources, Validation, Writing – review & editing, Funding acquisition, Supervision.

## Declaration of Competing Interest

None declared.
